# Supplementary material for: Association of measured quality with financial health among U.S. hospitals
Source: PLoS One. 2022 Apr 20;17(4):e0266696. doi: 10.1371/journal.pone.0266696 (PMC9020707; doi:10.1371/journal.pone.0266696)
Supplement: S1 File — (DOCX) [file pone.0266696.s001.docx]

**Supplemental Appendix**

**Table of Contents**

[S1 Table: Missing Data by Variable 2](#_Toc95572258)

[S1 Figure: Excluded Hospital Chart 3](#_Toc95572259)

[S2 Table: Multilevel Structure 4](#_Toc95572260)

[S3 Table: Years Contributed to Overall Sample 5](#_Toc95572261)

[eMethods 6](#_Toc95572262)

[S4 Table: Mixed effects Linear Regression Model for Association of Same Year Measured Quality and Operating Margin 7](#_Toc95572263)

[S5 Table: Mixed effects Linear Regression Model for Association of Same Year Measured Quality and Total Margin 8](#_Toc95572264)

[S6 Table: Mixed-effects Linear Regression for the Previous Year’s Measured Quality and Operating Margin 9](#_Toc95572265)

[S7 Table: Mixed-effects Linear Regression for the Association of Previous Year’s Measured Quality with Total Margin 10](#_Toc95572266)

[S8 Table: Mixed-effects Logistic Regression for the Same Year’s Measured Quality and Financial Distress 11](#_Toc95572267)

[S9 Table: Mixed-effects Logistic Regression for the Previous Year’s Measured Quality and Financial Distress 12](#_Toc95572268)

# S1 Table: Missing Data by Variable

| **Variable** | **Frequency** | **Total** | **% Missing** |
| --- | --- | --- | --- |
| Patient Safety Indicator-90 | 6,043 | 20,919 | 29% |
| Operating Margin | 1,262 | 20,919 | 6% |
| Total Margin | 1,258 | 20,919 | 6% |
| 30-day Readmission Rate | 610 | 20,919 | 3% |
| Rural | 530 | 20,919 | 3% |
| Financial Distress | 269 | 20,919 | 1% |
| X1: working capital to total assets | 259 | 20,919 | 1% |
| X2: retained earnings to total assets | 259 | 20,919 | 1% |
| X3: earnings before interest and taxes to total assets | 259 | 20,919 | 1% |
| X4: total equity to total liability | 259 | 20,919 | 1% |
| Year | 0 | 20,919 | 0% |
| Teaching Hospital | 0 | 20,919 | 0% |
| Hospital Bed Number | 0 | 20,919 | 0% |
| System Membership | 0 | 20,919 | 0% |
| Ownership | 0 | 20,919 | 0% |
| Medicare Payor Mix | 0 | 20,919 | 0% |
| Herfindahl-Hirschman Index | 0 | 20,919 | 0% |

**Source:** Authors’ analysis of Hospital Compare and American Hospital Association Survey, 2013-2018.

# S1 Figure: Excluded Hospital Chart

**
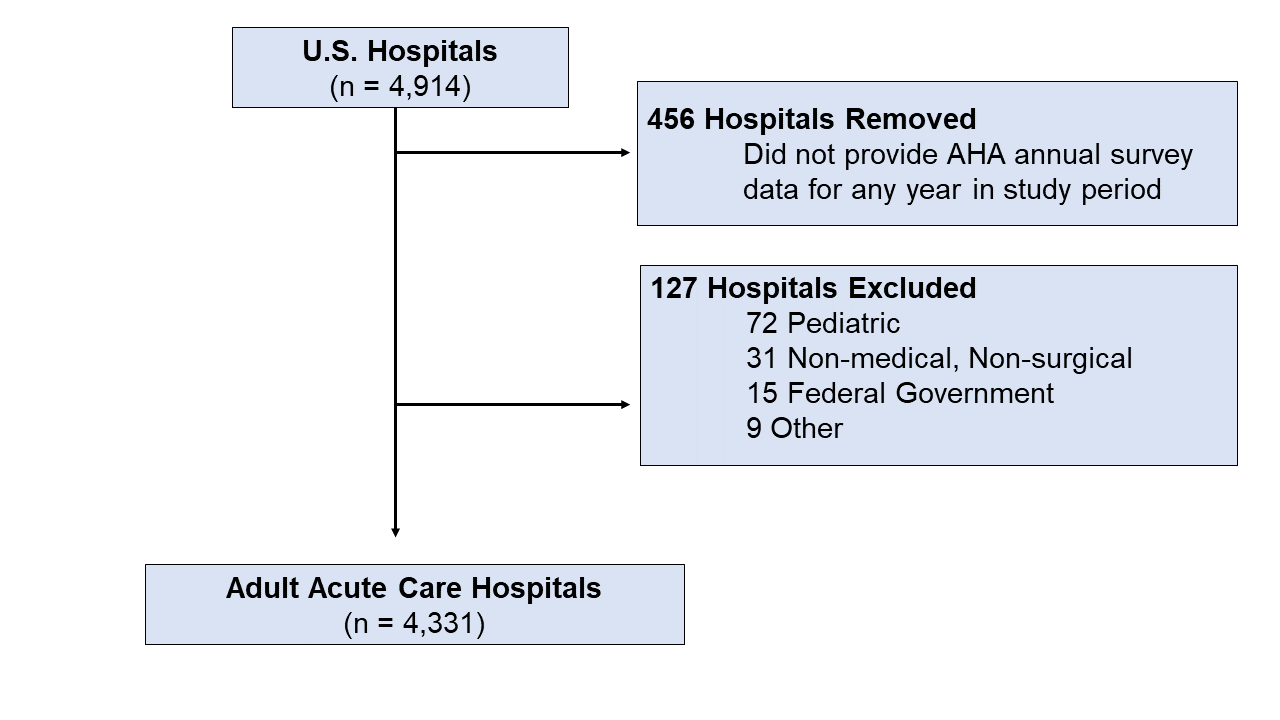
**

**Source:** Authors’ analysis of Hospital Compare and American Hospital Association Survey, 2013-2018.

# S2 Table: Multilevel Structure

| Level | Subindex | Variables |
| --- | --- | --- |
| Hospital (mcrnum) | j = 1, …, n | Rural, Teaching hospital status, number of hospital beds (categorical), system membership, ownership |
| Hospital-Observation (year) | t = 1, …, v_j_ | Dependent: operating margin, total margin, distress  Independent: 30-day readmission rate, patient-safety-indicator 90, Medicare payor mix, Herfindahl-Hirschman Index |

**Note:** mcrnum is the Medicare Provider Identification Number

# S3 Table: Years Contributed to Overall Sample

| **Pattern** | **Frequency** | **Percent** | **Observations** |
| --- | --- | --- | --- |
| 111111 | 2304 | 53.2 |  |
| 111101 | 195 | 4.5 |  |
| 111110 | 166 | 3.8 |  |
| 110000 | 156 | 3.6 |  |
| 111100 | 139 | 3.2 |  |
| 111011 | 113 | 2.6 |  |
| 100000 | 105 | 2.4 |  |
| 011111 | 93 | 2.2 |  |
| 001111 | 91 | 2.1 |  |
| Other Patterns | 969 | 22.4 |  |
| **Total** | 4331 | 100.0 | 20,919 |

**Source:** Authors’ analysis of Hospital Compare and American Hospital Association Survey, 2013-2018.

**Note:** "1" represents a year in which data were available.

"0" represents a year in which data were not available.

# eMethods

Altman Z-score: The modified Altman Z-score formula for financial distress is:

Z = 6.56*X1 + 3.26*X2 + 6.72*X3 + 1.05*X4 where:

X1 = working capital / total assets

X2 = retained earnings / total assets

X3 = earnings before interest and taxes / total assets

X4 = total equity / total liabilities.

We defined working capital as current assets minus current liabilities. Retained earnings was considered the cumulative retained earnings. Total equity was the extracted value from total fund balance.

Study Size: We did not perform a pre-analysis sample size calculation. We chose to incorporate all hospitals that met our inclusion criteria and had available data from the American Hospital Association and Hospital Compare.

Regression Analyses*:* We included operating margin and total margin as the dependent variables for the mixed effects linear regression. The hierarchical structure of the model with hospital-year observations clustered within hospitals was incorporated to account for correlation among observations from the same hospital. Our dependent variable was financial distress for the mixed effects logistic regression. We ran analyses with all independent variables of the same year as the dependent variable and with all independent variables lagged by one year. For our mixed effects generalized linear models, we estimated the following equation:

Operating Margin_i,j,_ = β_0_ + β_1_ Quality (quintile) + β_2_ Rural + β_3_ Teaching Hospital + β_4_ Number of Beds (grouped) + β_5_ System Membership + β_6_ Ownership + β_7_ Medicare Payor Mix + β_8_ Herfindahl-Hirschman + β_9_ Year + μ_i_ + μ_i,j_ + ε_i,j,k_

Operating Margin_i,j_ represents financial performance for the hospital-year i within hospital j. We included fixed year effects, random hospital (μ_j_) and residual error term (ε_i,j_). We did not include random slopes. The two quality variables were 30-day hospital-wide readmission rate and the Patient Safety Indicator-90 Score, and the reference group for each was the highest quintile (worst-performing). Separate models were run for each quality variable. Similar equations were estimated with the same independent variables and total margin as the dependent variable, and a mixed effects logistic regression was estimated with financial distress as the dependent variable. We used maximum likelihood estimation and, given that we did not assume a correlation among random effects, used unstructured covariance structure. The following STATA command was used:

meglm [depvar] ib(last).[quality_variable_quintile] i.c_year i.rural i.teach_hosp i.bed_groups i.sys_membership i.ownership c.medicare_share i.hhi_groups || mcrnum: , covariance(unstructured)

We constructed the conditional R^2^and marginal R^2^ values based on the work of Nakagawa et al. 2013.

For the continuous outcomes, the following equations were used to create these values:

Conditional R^2^ = (variance of fixed effects) / (variance of fixed effects + random variance + residual variance)

Marginal R^2^ = (variance of fixed effects + random variance) / (variance of fixed effects + random variance + residual variance).

For the dichotomous outcome of financial distress, the following equations were used:

Conditional R^2^ = (variance of fixed effects) / (variance of fixed effects + random variance + (π ^2)/3))

Marginal R^2^ = (variance of fixed effects + random variance) / (variance of fixed effects + random variance + (π ^2)/3)

# S4 Table: Mixed effects Linear Regression Model for Association of Same Year Measured Quality and Operating Margin

|  | **Null Model** |  | **Conditional^a^ (Readmission)** |  | **Conditional^b^**  **(PSI-90)** |  |
| --- | --- | --- | --- | --- | --- | --- |
| **Hospitals** | 4,186 |  | 4,081 |  | 2,920 |  |
| **Hospital-Years** | 19,657 |  | 18,687 |  | 13,387 |  |
| **Variable** | Estimate (95% CI) | P | Estimate (95% CI) | P | Estimate (95% CI) | P Value |
| 30-day Readmission Rate |  |  |  |  |  |  |
| Lowest Quintile | - | - | 0.95 (0.51 - 1.39) | < .001 | - | - |
| Second Quintile | - | - | 0.63 (0.21 - 1.04) | 0.003 | - | - |
| Third Quintile | - | - | 0.34 (-0.06 - 0.74) | 0.10 | - | - |
| Fourth Quintile | - | - | 0.16 (-0.22 - 0.55) | 0.41 | - | - |
| Highest Quintile (ref.) (reference) | - | - | Reference | NA | - | - |
| Patient Safety Indicator-90 |  |  |  |  |  |  |
| Lowest Quintile | - | - | - | - | 0.62 (0.17 - 1.08) | 0.007 |
| Second Quintile | - | - | - | - | 0.35 (-0.09 - 0.79) | 0.12 |
| Third Quintile | - | - | - | - | -0.1 (-0.55 - 0.36) | 0.67 |
| Fourth Quintile | - | - | - | - | 0.08 (-0.34 - 0.5) | 0.72 |
| Highest Quintile (ref.) (reference) | - | - | - | - | Reference | NA |
| Year |  |  |  |  |  |  |
| 2014 | - | - | 1.3 (0.96 - 1.64) | < .001 | 1.72 (1.33 - 2.12) | < .001 |
| 2015 | - | - | 1.6 (1.26 - 1.94) | < .001 | 1.92 (1.52 - 2.32) | < .001 |
| 2016 | - | - | 0.45 (0.1 - 0.8) | 0.01 | 0.89 (0.48 - 1.3) | < .001 |
| 2017 | - | - | -0.45 (-0.81 - -0.09) | 0.01 | 0.003 (-0.43 - 0.42) | 0.99 |
| 2018 | - | - | 0.12 (-0.23 - 0.48) | 0.496 | 0.7 (0.28 - 1.12) | 0.001 |
| Rural | - | - | -1.78 (-2.48 - -1.09) | < .001 | -2.03 (-2.91 - -1.16) | < .001 |
| Teaching Hospital | - | - | 0.52 (0.06 - 0.97) | 0.025 | 0.22 (-0.28 - 0.73) | 0.38 |
| Number of Beds |  |  |  |  |  |  |
| 1-99 (Reference) | - | - | Reference | NA | Reference | NA |
| 100-299 | - | - | 2.1 (1.43 - 2.77) | < .001 | 2.27 (1.52 - 3.02) | < .001 |
| 300 or more | - | - | 2.26 (1.36 - 3.17) | < .001 | 2.42 (1.43 - 3.41) | < .001 |
| System Membership | - | - | 1.78 (1.23 - 2.33) | < .001 | 1.6 (0.94 - 2.27) | < .001 |
| Ownership |  |  |  |  |  |  |
| Non-Profit (reference) | - | - | Reference | NA | Reference | NA |
| For-Profit | - | - | 3.67 (2.85 - 4.5) | < .001 | 4.25 (3.36 - 5.14) | < .001 |
| Government (non-federal) | - | - | -5.17 (-5.97 to  -4.36) | < .001 | -4.93 (-6.01 to  -3.85) | < .001 |
| Medicare Payor Mix | - | - | 0.01 (0 - 0.02) | 0.01 | 0.01 (0 - 0.03) | 0.003 |
| Herfindahl-Hirschman Index |  |  |  |  |  |  |
| Low Concentration (reference) | - | - | Reference | NA | Reference | NA |
| Moderate Concentration | - | - | 0.84 (0.34 - 1.34) | 0.001 | 0.87 (0.29 - 1.45) | 0.003 |
| High Concentration | - | - | 0.64 (0.05 - 1.22) | 0.032 | 0.55 (-0.16 - 1.27) | 0.13 |
| Fixed effect Intercept | -3.43 | - | -6.40 |  | -6.32 |  |
| Random hospital intercept, variance | 142.2 | - | 116.5 |  | 123.1 |  |
| Residuals, variance | 45.7 | - | 43.5 |  | 41.6 |  |
| Marginal R^2^ | - | - | 0.095 |  | 0.075 |  |
| Conditional R^2^ | - | - | 0.754 |  | 0.767 |  |
| Akaike Information Criteria | 142085 | - | 133767 |  | 95520 |  |
| Bayesian Information Criteria | 142109 | - | 133939 |  | 95685 |  |

a Conditional model included random hospital intercepts and fixed effects for the year, quality (readmission rate), and the following hospital characteristics: rural, teaching status, bed size, hospital system membership, ownership, Medicare payor mix, and Herfindahl-Hirschman Index

b Conditional model with the same fixed and random effects but PSI-90 score in place of 30-day Readmission Rate.

# S5 Table: Mixed effects Linear Regression Model for Association of Same Year Measured Quality and Total Margin

|  | **Null Model** |  | **Conditional^a^ (Readmission)** |  | **Conditional^b^**  **(PSI-90)** |  |
| --- | --- | --- | --- | --- | --- | --- |
| **Hospitals** | 4,209 |  | 4,094 |  | 2,928 |  |
| **Hospital-Years** | 19,661 |  | 18,653 |  | 13,375 |  |
| **Variable** | Estimate (95% CI) | P | Estimate (95% CI) | P | Estimate (95% CI) | P Value |
| 30-day Readmission Rate |  |  |  |  |  |  |
| Lowest Quintile | - | - | 0.77 (0.42 - 1.12) | < .001 | - | - |
| Second Quintile | - | - | 0.54 (0.21 - 0.87) | 0.001 | - | - |
| Third Quintile | - | - | 0.38 (0.06 - 0.7) | 0.02 | - | - |
| Fourth Quintile | - | - | 0.23 (-0.08 - 0.54) | 0.14 | - | - |
| Highest Quintile (ref.) (reference) | - | - | - | - | - | - |
| Patient Safety Indicator-90 |  |  |  |  |  |  |
| Lowest Quintile | - | - | - | - | 0.52 (0.16 - 0.89) | 0.004 |
| Second Quintile | - | - | - | - | 0.26 (-0.09 - 0.62) | 0.15 |
| Third Quintile | - | - | - | - | -0.07 (-0.43 - 0.3) | 0.72 |
| Fourth Quintile | - | - | - | - | 0.11 (-0.22 - 0.45) | 0.51 |
| Highest Quintile (ref.) (reference) | - | - | - | - | - | - |
| Year |  |  |  |  |  |  |
| 2014 | - | - | 0.17 (-0.1 - 0.44) | 0.21 | 0.3 (-0.01 - 0.62) | 0.06 |
| 2015 | - | - | -0.07 (-0.34 - 0.21) | 0.64 | -0.2 (-0.52 - 0.12) | 0.22 |
| 2016 | - | - | -0.46 (-0.74 - -0.18) | 0.001 | -0.38 (-0.71 - -0.05) | 0.02 |
| 2017 | - | - | -0.93 (-1.22 - -0.64) | < .001 | -0.85 (-1.19 - -0.51) | < .001 |
| 2018 | - | - | -0.89 (-1.17 - -0.6) | < .001 | -0.81 (-1.15 - -0.47) | < .001 |
| Rural | - | - | -1.23 (-1.72 - -0.73) | < .001 | -1.43 (-2.06 - -0.79) | < .001 |
| Teaching Hospital | - | - | 0.51 (0.17 - 0.86) | 0.004 | 0.37 (-0.02 - 0.76) | 0.06 |
| Number of Beds |  |  |  |  |  |  |
| 1-99 (Reference) | - | - |  | NA |  |  |
| 100-299 | - | - | 0.63 (0.15 - 1.12) | 0.010 | 0.68 (0.12 - 1.24) | 0.02 |
| 300 or more | - | - | 1.47 (0.81 - 2.12) | < .001 | 1.41 (0.67 - 2.14) | < .001 |
| System Membership | - | - | 0.57 (0.17 - 0.98) | 0.006 | 0.31 (-0.19 - 0.81) | 0.22 |
| Ownership |  |  |  |  |  |  |
| Non-Profit (reference) | - | - |  |  |  |  |
| For-Profit | - | - | 1.35 (0.76 - 1.94) | < .001 | 1.55 (0.89 - 2.2) | < .001 |
| Government (non-federal) | - | - | -1.1 (-1.67 - -0.54) | < .001 | -1.14 (-1.92 - -0.36) | 0.004 |
| Medicare Payor Mix | - | - | 0.01 (0 - 0.01) | 0.05 | 0 (-0.02 - 0.01) | 0.50 |
| Herfindahl-Hirschman Index |  |  |  |  |  |  |
| Low Concentration (reference) | - | - |  |  |  |  |
| Moderate Concentration | - | - | 0.61 (0.23 - 0.99) | 0.001 | 0.41 (-0.03 - 0.85) | 0.07 |
| High Concentration | - | - | 0.56 (0.13 - 0.98) | 0.010 | 0.3 (-0.23 - 0.83) | 0.26 |
| Fixed effect Intercept | 4.80 |  | 3.56 |  | 4.78 |  |
| Random hospital intercept, variance | 52.8 |  | 49.8 |  | 57.0 |  |
| Residuals, variance | 28.8 |  | 28.0 |  | 26.7 |  |
| Marginal R^2^ | - |  | 0.03 |  | 0.03 |  |
| Conditional R^2^ | - |  | 0.65 |  | 0.69 |  |
| Akaike Information Criteria | 131060 |  | 123849 |  | 88685 |  |
| Bayesian Information Criteria | 131084 |  | 124022 |  | 88850 |  |

a Conditional model included random hospital intercepts and fixed effects for the year, quality (readmission rate), and the following hospital characteristics: rural, teaching status, bed size, hospital system membership, ownership, Medicare payor mix, and Herfindahl-Hirschman Index

b Conditional model with the same fixed and random effects but PSI-90 score in place of 30-day Readmission Rate.

# S6 Table: Mixed-effects Linear Regression for the Previous Year’s Measured Quality and Operating Margin

|  | **Null Model** |  | **Conditional^a^ (Readmission)** |  | **Conditional^b^**  **(PSI-90)** |  |
| --- | --- | --- | --- | --- | --- | --- |
| **Hospitals** | 4,186 |  | 3,722 |  | 2,690 |  |
| **Hospital-Years** | 19,657 |  | 14,006 |  | 10,047 |  |
| **Variable** | Estimate (95% CI) | P | Estimate (95% CI) | P | Estimate (95% CI) | P Value |
| 30-day Readmission Rate |  |  |  |  |  |  |
| Lowest Quintile |  |  |  |  |  |  |
| Second Quintile | - | - | 0.91 (0.41 - 1.4) | < .001 | - | - |
| Third Quintile | - | - | 0.56 (0.09 - 1.02) | 0.02 | - | - |
| Fourth Quintile | - | - | 0.38 (-0.07 - 0.83) | 0.10 | - | - |
| Highest Quintile (ref.) (reference) | - | - | 0.43 (0 - 0.86) | 0.05 | - | - |
| Patient Safety Indicator-90 | - | - | - | - | - | - |
| Lowest Quintile |  |  |  |  |  |  |
| Second Quintile | - | - | - | - | 0.66 (0.15 - 1.16) | 0.01 |
| Third Quintile | - | - | - | - | 0.33 (-0.16 - 0.82) | 0.19 |
| Fourth Quintile | - | - | - | - | -0.02 (-0.53 - 0.48) | 0.92 |
| Highest Quintile (ref.) (reference) | - | - | - | - | 0.36 (-0.11 - 0.82) | 0.13 |
| Year | - | - | - | - | - | - |
| 2014 | - | - | 0.32 (-0.01 - 0.65) | 0.06 | 0.30 (-0.09 - 0.69) | 0.13 |
| 2015 | - | - | -0.84 (-1.18 - -0.5) | < .001 | -0.73 (-1.13 - -0.33) | < .001 |
| 2016 | - | - | -1.82 (-2.17 - -1.46) | < .001 | -1.74 (-2.15 - -1.32) | < .001 |
| 2017 | - | - | -1.26 (-1.61 - -0.9) | < .001 | -1.05 (-1.47 - -0.63) | < .001 |
| Rural | - | - | -2.24 (-3 - -1.48) | < .001 | -2.42 (-3.36 - -1.47) | < .001 |
| Teaching Hospital | - | - | 0.30 (-0.23 - 0.82) | 0.27 | -0.05 (-0.62 - 0.53) | 0.87 |
| Number of Beds |  |  |  |  |  |  |
| 1-99 (Reference) | - | - |  |  |  |  |
| 100-299 | - | - | 2.03 (1.28 - 2.78) | < .001 | 1.96 (1.12 - 2.8) | < .001 |
| 300 or more | - | - | 1.99 (0.99 - 2.99) | < .001 | 1.98 (0.89 - 3.06) | < .001 |
| System Membership | - | - | 2.16 (1.54 - 2.78) | < .001 | 2.19 (1.44 - 2.93) | < .001 |
| Ownership |  |  |  |  |  |  |
| Non-Profit (reference) | - | - |  |  |  |  |
| For-Profit | - | - | 3.45 (2.5 - 4.4) | < .001 | 4.2 (3.17 - 5.23) | < .001 |
| Government (non-federal) | - | - | -5.22 (-6.12 - -4.33) | < .001 | -5.27 (-6.47 - -4.07) | < .001 |
| Medicare Payor Mix | - | - | 0.02 (0 - 0.03) | 0.004 | 0.01 (0 - 0.03) | 0.14 |
| Herfindahl-Hirschman Index |  |  |  |  |  |  |
| Low Concentration (reference) | - | - |  |  |  |  |
| Moderate Concentration | - | - | 0.52 (-0.02 - 1.06) | 0.06 | 0.59 (-0.04 - 1.21) | 0.067 |
| High Concentration | - | - | 0.25 (-0.39 - 0.88) | 0.45 | 0.17 (-0.6 - 0.94) | 0.664 |
| Fixed effect Intercept | -3.43 |  | -4.62 |  | -4.22 |  |
| Random hospital intercept, variance | 142.2 |  | 119.2 |  | 125.8 |  |
| Residuals, variance | 45.7 |  | 37.8 |  | 35.8 |  |
| Marginal R^2^ | - |  | 0.10 |  | 0.08 |  |
| Conditional R^2^ | - |  | 0.78 |  | 0.80 |  |
| Akaike Information Criteria | 142085 |  | 99846 |  | 71400 |  |
| Bayesian Information Criteria | 142109 |  | 100004 |  | 71551 |  |

a Conditional model included random hospital intercepts and fixed effects for the year, quality (readmission rate), and the following hospital characteristics: rural, teaching status, bed size, hospital system membership, ownership, Medicare payor mix, and Herfindahl-Hirschman Index

b Conditional model with the same fixed and random effects but PSI-90 score in place of 30-day Readmission Rate.

# S7 Table: Mixed-effects Linear Regression for the Association of Previous Year’s Measured Quality with Total Margin

|  | **Null Model** |  | **Conditional^a^ (Readmission)** |  | **Conditional^b^**  **(PSI-90)** |  |
| --- | --- | --- | --- | --- | --- | --- |
| **Hospitals** | 4,209 |  | 3,740 |  | 2,700 |  |
| **Hospital-Years** | 19,661 |  | 13,992 |  | 10,051 |  |
| **Variable** | Estimate (95% CI) | P | Estimate (95% CI) | P | Estimate (95% CI) | P Value |
| 30-day Readmission Rate |  |  |  |  |  |  |
| Lowest Quintile | - | - | 0.77 (0.37 - 1.16) | < .001 | - | - |
| Second Quintile | - | - | 0.7 (0.33 - 1.07) | < .001 | - | - |
| Third Quintile | - | - | 0.59 (0.23 - 0.95) | 0.001 | - | - |
| Fourth Quintile | - | - | 0.38 (0.03 - 0.73) | 0.03 | - | - |
| Highest Quintile (ref.) (reference) | - | - | - | - | - | - |
| Patient Safety Indicator-90 |  |  |  |  |  |  |
| Lowest Quintile | - | - | - | - | 0.42 (0.01 - 0.82) | 0.04 |
| Second Quintile | - | - | - | - | 0.24 (-0.15 - 0.64) | 0.23 |
| Third Quintile | - | - | - | - | -0.07 (-0.47 - 0.34) | 0.75 |
| Fourth Quintile | - | - | - | - | 0.14 (-0.23 - 0.52) | 0.45 |
| Highest Quintile (ref.) (reference) | - | - | - | - | - | - |
| Year |  |  |  |  |  |  |
| 2014 | - | - | -0.37 (-0.64 - -0.1) | 0.007 | -0.67 (-0.99 - -0.35) | < .001 |
| 2015 | - | - | -0.70 (-0.98 - -0.42) | < .001 | -0.80 (-1.12 - -0.47) | < .001 |
| 2016 | - | - | -1.2 (-1.49 - -0.92) | < .001 | -1.33 (-1.67 - -0.99) | < .001 |
| 2017 | - | - | -1.23 (-1.52 - -0.94) | < .001 | -1.32 (-1.66 - -0.98) | < .001 |
| Rural | - | - | -1.5 (-2.04 - -0.95) | < .001 | -1.78 (-2.47 - -1.09) | < .001 |
| Teaching Hospital | - | - | 0.31 (-0.1 - 0.72) | 0.14 | 0.21 (-0.24 - 0.66) | 0.35 |
| Number of Beds |  |  |  |  |  |  |
| 1-99 (Reference) | - | - |  |  |  |  |
| 100-299 | - | - | 0.48 (-0.06 - 1.03) | 0.08 | 0.34 (-0.29 - 0.97) | 0.28 |
| 300 or more | - | - | 1.14 (0.41 - 1.86) | 0.002 | 0.87 (0.06 - 1.68) | 0.04 |
| System Membership | - | - | 0.88 (0.42 - 1.34) | < .001 | 0.65 (0.09 - 1.22) | 0.02 |
| Ownership |  |  |  |  |  |  |
| Non-Profit (reference) | - | - |  |  |  |  |
| For-Profit | - | - | 1.24 (0.57 - 1.92) | < .001 | 1.58 (0.82 - 2.33) | < .001 |
| Government (non-federal) | - | - | -1.35 (-1.98 - -0.72) | < .001 | -1.72 (-2.58 - -0.86) | < .001 |
| Medicare Payor Mix | - | - | 0 (0 - 0.01) | 0.42 | -0.01 (-0.02 - 0) | 0.14 |
| Herfindahl-Hirschman Index |  |  |  |  |  |  |
| Low Concentration (reference) | - | - |  |  |  |  |
| Moderate Concentration | - | - | -0.01 (-0.42 - 0.41) | 0.98 | -0.07 (-0.56 - 0.41) | 0.78 |
| High Concentration | - | - | 0.11 (-0.36 - 0.58) | 0.64 | -0.17 (-0.75 - 0.4) | 0.56 |
| Fixed effect Intercept | 4.80 |  | 4.56 |  | 6.27 |  |
| Random hospital intercept, variance | 52.8 |  | 52.3 |  | 59.3 |  |
| Residuals, variance | 28.8 |  | 25.1 |  | 23.8 |  |
| Marginal R^2^ | - |  | 0.04 |  | 0.032 |  |
| Conditional R^2^ | - |  | 0.69 |  | .723 |  |
| Akaike Information Criteria | 131060 |  | 92674 |  | 66497 |  |
| Bayesian Information Criteria | 131084 |  | 92832 |  | 66649 |  |

a Conditional model included random hospital intercepts and fixed effects for the year, quality (readmission rate), and the following hospital characteristics: rural, teaching status, bed size, hospital system membership, ownership, Medicare payor mix, and Herfindahl-Hirschman Index

b Conditional model with the same fixed and random effects but PSI-90 score in place of 30-day Readmission Rate.

# S8 Table: Mixed-effects Logistic Regression for the Same Year’s Measured Quality and Financial Distress

|  | **Null Model** |  | **Conditional^a^ (Readmission)** |  | **Conditional^b^**  **(PSI-90)** |  |
| --- | --- | --- | --- | --- | --- | --- |
| **Hospitals** | 4,282 |  | 4,163 |  | 2,997 |  |
| **Hospital-Years** | 20,650 |  | 19,539 |  | 14,138 |  |
| **Variable** | Estimate (95% CI) | P | Estimate (95% CI) | P | Estimate (95% CI) | P Value |
| 30-day Readmission Rate |  |  |  |  |  |  |
| Lowest Quintile | - | - | 0.56 (0.45 - 0.7) | < .001 | - | - |
| Second Quintile | - | - | 0.79 (0.64 - 0.97) | 0.022 | - | - |
| Third Quintile | - | - | 0.79 (0.65 - 0.97) | 0.022 | - | - |
| Fourth Quintile | - | - | 0.94 (0.78 - 1.13) | 0.51 | - | - |
| Highest Quintile (ref.) (reference) | - | - | - | - | - | - |
| Patient Safety Indicator-90 |  |  |  |  |  |  |
| Lowest Quintile | - | - | - | - | 0.70 (0.56 - 0.89) | 0.003 |
| Second Quintile | - | - | - | - | 0.79 (0.63 - 0.99) | 0.04 |
| Third Quintile | - | - | - | - | 0.99 (0.79 - 1.25) | 0.95 |
| Fourth Quintile | - | - | - | - | 0.96 (0.77 - 1.19) | 0.70 |
| Highest Quintile (ref.) (reference) | - | - | - | - | - | - |
| Year |  |  |  |  |  |  |
| 2014 | - | - | 1.11 (0.92 - 1.33) | 0.29 | 1.07 (0.86 - 1.32) | 0.564 |
| 2015 | - | - | 1.32 (1.09 - 1.59) | 0.004 | 1.42 (1.15 - 1.77) | 0.001 |
| 2016 | - | - | 1.54 (1.27 - 1.86) | < .001 | 1.41 (1.13 - 1.75) | 0.002 |
| 2017 | - | - | 1.59 (1.31 - 1.93) | < .001 | 1.36 (1.08 - 1.71) | 0.008 |
| 2018 | - | - | 2.07 (1.71 - 2.51) | < .001 | 2.03 (1.63 - 2.53) | < .001 |
| Rural | - | - | 0.64 (0.5 - 0.82) | < .001 | 0.82 (0.62 - 1.1) | 0.19 |
| Teaching Hospital | - | - | 0.86 (0.7 - 1.06) | 0.15 | 0.95 (0.76 - 1.19) | 0.67 |
| Number of Beds |  |  |  |  |  |  |
| 1-99 (Reference) | - | - |  |  |  |  |
| 100-299 | - | - | 0.65 (0.5 - 0.83) | 0.001 | 0.56 (0.43 - 0.73) | < .001 |
| 300 or more | - | - | 0.46 (0.33 - 0.65) | < .001 | 0.37 (0.26 - 0.53) | < .001 |
| System Membership | - | - | 1.73 (1.39 - 2.14) | < .001 | 1.92 (1.49 - 2.47) | < .001 |
| Ownership |  |  |  |  |  |  |
| Non-Profit (reference) | - | - |  |  |  |  |
| For-Profit | - | - | 4.53 (3.43 - 5.98) | < .001 | 3.64 (2.74 - 4.84) | < .001 |
| Government (non-federal) | - | - | 1.45 (1.1 - 1.91) | 0.008 | 1.9 (1.35 - 2.69) | < .001 |
| Medicare Payor Mix | - | - | 1 (0.99 - 1) | 0.15 | 0.99 (0.99 - 1) | 0.01 |
| Herfindahl-Hirschman Index |  |  |  |  |  |  |
| Low Concentration (reference) | - | - |  |  |  |  |
| Moderate Concentration | - | - | 0.87 (0.71 - 1.07) | 0.20 | 0.85 (0.67 - 1.08) | 0.19 |
| High Concentration | - | - | 0.85 (0.68 - 1.06) | 0.14 | 0.84 (0.65 - 1.08) | 0.17 |
| Fixed effect Intercept | -3.01 |  | -2.96 |  | -2.62 |  |
| Random hospital intercept, variance | 11.8 |  | 10.6 |  | 8.978 |  |
| Marginal R^2^ | - |  | 0.04 |  | 0.05 |  |
| Conditional R^2^ | - |  | 0.77 |  | 0.74 |  |
| Akaike Information Criteria | 15654 |  | 14590 |  | 10959 |  |
| Bayesian Information Criteria | 15670 |  | 14755 |  | 11118 |  |

a Conditional model included random hospital intercepts and fixed effects for the year, quality (readmission rate), and the following hospital characteristics: rural, teaching status, bed size, hospital system membership, ownership, Medicare payor mix, and Herfindahl-Hirschman Index

b Conditional model with the same fixed and random effects but PSI-90 score in place of 30-day Readmission Rate.

# S9 Table: Mixed-effects Logistic Regression for the Previous Year’s Measured Quality and Financial Distress

|  | **Null Model** |  | **Conditional^a^ (Readmission)** |  | **Conditional^b^**  **(PSI-90)** |  |
| --- | --- | --- | --- | --- | --- | --- |
| **Hospitals** | 4,282 |  | 3,799 |  | 2,759 |  |
| **Hospital-Years** | 20,650 |  | 14,627 |  | 10,587 |  |
| **Variable** | Estimate (95% CI) | P | Estimate (95% CI) | P | Estimate (95% CI) | P Value |
| 30-day Readmission Rate |  |  |  |  |  |  |
| Lowest Quintile | - | - | 0.65 (0.5 - 0.84) | 0.001 | - | - |
| Second Quintile | - | - | 0.67 (0.53 - 0.86) | 0.001 | - | - |
| Third Quintile | - | - | 0.76 (0.6 - 0.96) | 0.024 | - | - |
| Fourth Quintile | - | - | 0.87 (0.7 - 1.1) | 0.25 | - | - |
| Highest Quintile (ref.) (reference) | - | - | - | - | - | - |
| Patient Safety Indicator-90 |  |  |  |  |  |  |
| Lowest Quintile | - | - | - | - | 0.65 (0.49 - 0.85) | 0.002 |
| Second Quintile | - | - | - | - | 0.73 (0.56 - 0.95) | 0.02 |
| Third Quintile | - | - | - | - | 0.84 (0.64 - 1.1) | 0.21 |
| Fourth Quintile | - | - | - | - | 0.87 (0.68 - 1.12) | 0.28 |
| Highest Quintile (ref.) (reference) | - | - | - | - | - | - |
| Year |  |  |  |  |  |  |
| 2014 | - | - | 1.23 (1.01 - 1.5) | 0.04 | 1.41 (1.12 - 1.77) | 0.003 |
| 2015 | - | - | 1.45 (1.18 - 1.78) | < .001 | 1.42 (1.12 - 1.79) | 0.003 |
| 2016 | - | - | 1.45 (1.18 - 1.79) | < .001 | 1.32 (1.04 - 1.68) | 0.02 |
| 2017 | - | - | 1.91 (1.55 - 2.35) | < .001 | 1.92 (1.51 - 2.44) | < .001 |
| Rural | - | - | 0.73 (0.55 - 0.96) | 0.02 | 0.92 (0.66 - 1.27) | 0.60 |
| Teaching Hospital | - | - | 0.97 (0.76 - 1.24) | 0.81 | 1.09 (0.84 - 1.41) | 0.50 |
| Number of Beds |  |  |  |  |  |  |
| 1-99 (Reference) | - | - |  |  |  |  |
| 100-299 | - | - | 0.69 (0.52 - 0.91) | 0.009 | 0.61 (0.45 - 0.82) | 0.001 |
| 300 or more | - | - | 0.45 (0.3 - 0.65) | < .001 | 0.36 (0.24 - 0.53) | < .001 |
| System Membership | - | - | 1.42 (1.11 - 1.81) | 0.005 | 1.6 (1.21 - 2.13) | 0.001 |
| Ownership |  |  |  |  |  |  |
| Non-Profit (reference) | - | - |  |  |  |  |
| For-Profit | - | - | 4.48 (3.23 - 6.2) | < .001 | 3.49 (2.52 - 4.84) | < .001 |
| Government (non-federal) | - | - | 1.27 (0.93 - 1.74) | 0.13 | 1.61 (1.1 - 2.37) | 0.02 |
| Medicare Payor Mix | - | - | 0.99 (0.99 - 1) | 0.04 | 0.99 (0.98 - 1) | 0.01 |
| Herfindahl-Hirschman Index |  |  |  |  |  |  |
| Low Concentration (reference) | - | - |  |  |  |  |
| Moderate Concentration | - | - | 1.03 (0.81 - 1.3) | 0.83 | 0.90 (0.69 - 1.18) | 0.45 |
| High Concentration | - | - | 1.03 (0.8 - 1.32) | 0.83 | 0.93 (0.7 - 1.23) | 0.62 |
| Fixed effect Intercept | -3.01 |  | 0.05 |  | 0.08 |  |
| Random hospital intercept, variance | 11.8 |  | 11.6 |  | 9.04 |  |
| Marginal R^2^ | - |  | 0.030 |  | 0.039 |  |
| Conditional R^2^ | - |  | 0.785 |  | 0.743 |  |
| Akaike Information Criteria | 15654 |  | 11139 |  | 8396 |  |
| Bayesian Information Criteria | 15670 |  | 11291 |  | 8541 |  |

a Conditional model included random hospital intercepts and fixed effects for the year, quality (readmission rate), and the following hospital characteristics: rural, teaching status, bed size, hospital system membership, ownership, Medicare payor mix, and Herfindahl-Hirschman Index

b Conditional model with the same fixed and random effects but PSI-90 score in place of 30-day Readmission Rate.
